# Development of a Physical Therapy Telehealth Examination Battery for People with Parkinson Disease

**DOI:** 10.5195/ijt.2023.6592

**Published:** 2023-12-12

**Authors:** Chelsea E. Macpherson, Julie Fineman, Anuja Chandrana, Lori Quinn

**Affiliations:** 1 Department of Biobehavioral Sciences, Teachers College, Columbia University, New York, NY, USA; 2 Doctor of Physical Therapy Program, Marist College, Poughkeepsie, NY, USA; 3 Department of Rehabilitation and Regenerative Medicine (Physical Therapy), Columbia University Irving Medical Center, New York, NY, USA

**Keywords:** Algorithm (Decision tree), Examination, Parkinson disease, Physical therapy, Telehealth

## Abstract

**Scope:**

The rapid transition to telehealth following the COVID-19 pandemic raised challenges for remote delivery of physical therapy. One challenge was identifying outcome measures for people with Parkinson Disease (PwP) that could safely be conducted via telehealth. This paper evaluates the feasibility of a telehealth physical therapy examination battery for PwP in early to middle stage of disease progression.

**Methodology:**

We reviewed recommended outcome measures from the American Physical Therapy Association's Academy of Neurologic Physical Therapy (ANPT) Parkinson Evidence Database to Guide Effectiveness (EDGE) document and evaluated their appropriateness for remote administration. A clinical decision tree was created to streamline the examination process, incorporating elements of the ANPT movement analysis of tasks as a movement screen. The examination battery was then conducted on three PwP and evaluated for safety and feasibility.

**Conclusion:**

This physical therapy telehealth examination battery provides physical therapists with a method to conduct safe and efficient remote assessments for PwP.

Parkinson disease (PD) is a neurodegenerative disease that results in a range of motor symptoms, including tremor, bradykinesia, rigidity, and postural instability, as well as a variety of non-motor symptoms (Beitz, 2014). Over 8 million people worldwide are currently living with PD, and that number is expected to rise with increased access to healthcare and the combination of genetic and environmental factors (Dorsey et al., 2018). The progressive nature of PD requires management of the disease over many years (Mak et al., 2017), and physical therapy plays a crucial role in managing functional decline, preventing secondary complications, and potentially slowing disease progression through exercise and other lifestyle modifications (Ellis et al., 2021; Radder et al., 2017). Throughout the course of the disease people with Parkinson Disease (PwP) should engage in both consultative and restorative physical therapy. Consultative, or proactive, physical therapy occurs soon after diagnosis for assessment, treatment of early deficits, and promotion of meaningful activities, whereas restorative rehabilitation focuses on promoting functional improvements when more significant motor impairments are involved (Rafferty et al., 2021). Both of these pathways, but in particular consultative physical therapy, are amenable to being delivered by telehealth, providing an opportunity for improved access to services and specialist care across the stages of the disease (Rafferty et al., 2021).

The COVID-19 pandemic forced many aspects of healthcare to transition to telehealth to best serve our communities. Physical therapy is one discipline that rapidly transitioned to a telehealth model of care, and there is continued interest from patients and clinicians to deliver some forms of assessment and intervention via telehealth (Buabbas et al., 2022, Quinn et al., 2020). Physical therapy delivered via telehealth improves patient satisfaction (Tenforde et al., 2020) and compliance (Harrell et al., 2022; Lai et al., 2020). Benefits of telehealth over in-person therapy include providing continuity of care and improving access to people in rural locations, for those with mobility deficits, limited care-partner support, financial support, and transportation issues, among others (Colón-Semenza et al., 2023; Seron et al., 2021).

Several studies have supported the feasibility and efficacy of telehealth-delivered interventions for PwP (Shih et al., 2022; Vellata et al., 2021). While researchers have used a range of outcome assessments in these studies, there has not been a structured approach to conducting physical therapy assessments via telehealth for PwP. A systematic review in people with chronic conditions found that several performance-based measures of physical function may have sufficient reliability and criterion validity when administered via telehealth (Walsh et al., 2022). However, physical therapy assessments delivered via telehealth require special considerations, including technology literacy and availability, safety considerations and environmental setup (Zischke et al., 2021).

The purpose of the physical therapy examination is to gather data used to diagnose movement system problems in order to best direct interventions (*APTA Guide to Physical Therapy Practice 4.0*, 2023). Standardized outcome measures are typically used to evaluate change over time following physical therapy intervention. For best clinical practice, physical therapists may rely on the American Physical Therapy Association's Academy of Neurologic Physical Therapy (ANPT) Parkinson Evidence Database to Guide Effectiveness (PD-EDGE) (Kegelmeyer et al., n.d.) to select appropriate clinical outcome measures for PwP across disease stages (i.e., Hoehn & Yahr I-V), care settings, and levels of the WHO International Classification of Function (ICF). However, the PD-EDGE documents do not provide recommendations for telehealth. Although some items or measures may be readily adaptable, others cannot be automatically implemented within this setting (i.e., compensatory stepping reactions, 6-minute walk test). Furthermore, safety may be a concern when assessing balance or gait, especially in those with known balance impairments (i.e., Hoehn & Yahr III or greater).

The aim of this paper is to propose a physical therapy examination battery for use over telehealth for PwP. We used a novel decision tree process to select examination items and outcome measures that are individualized to each patient presentation. We further provide three case exemplars evaluating the feasibility of implementing this examination battery for PwP.

## Methods

### Development of Telehealth Examination Battery for PwP

#### Selection of Tests and Measures

**Figure 1.**
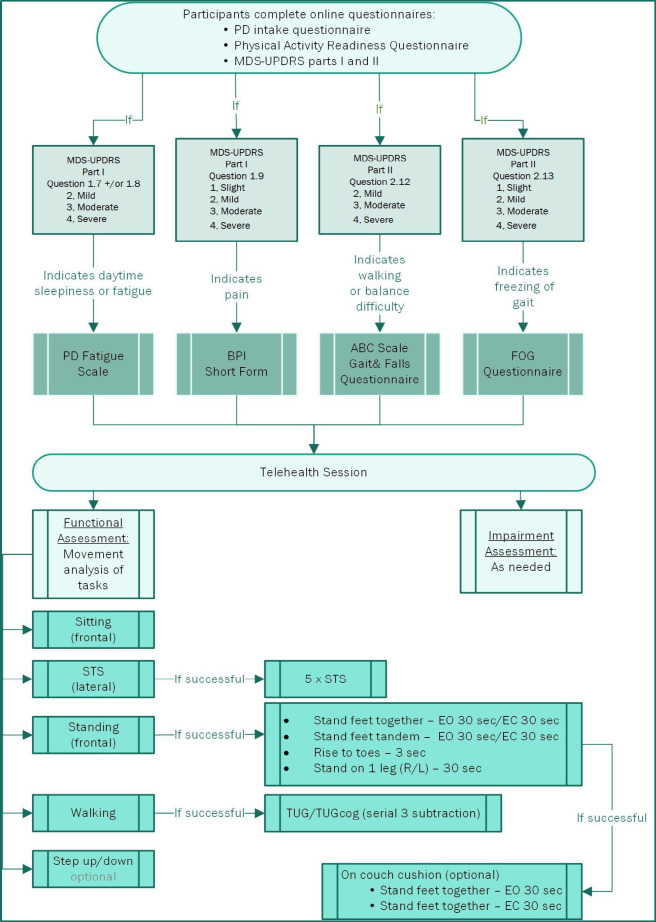
Overview of Clinical Decision Tree

We reviewed recommended measures from the ANPT PD EDGE documents, evaluated their relevance and appropriateness for telehealth examination (see Figure 1). We did not include the 6-minute walk test (Kobayashi et al., 2017), 10-meter walk test (Steffen & Seney, 2008), or several mini-Balance Evaluation Systems Test (Mini-BESTest) (King et al., 2012; Leddy et al., 2011) items due to environmental limitations and safety concerns. We chose not to include the Montreal Cognitive Assessment (MoCA) (Abdolahi et al., 2016) and the Parkinson Disease Questionnaire-8 (PDQ-8) (Dal Bello-Haas et al., 2011). Instead, we chose to focus on activity limitations and participation restrictions that were linked with motor system impairments, as these could easily be implemented in telehealth examinations.

We supplemented with additional outcome measures and examination items that could be conducted via telehealth. These included the Physical Activity Readiness Questionnaire (PAR-Q) (Thomas et al., 1992), Brief Pain Inventory (BPI) (Taghizadeh et al., 2022), movement analysis of tasks (Quinn et al., 2021) and several standing balance items.

#### Patient-reported Outcomes

Participants were sent questionnaires via a secure web link (via Project REDCap, Vanderbilt University, Nashville, TN, USA) to be completed prior to the telehealth session. We created a clinical decision tree (see Figure 1) with branching logic to streamline the examination process. The first questionnaires sent were an intake questionnaire regarding demographics, fall history and rehabilitation history; the Physical Activity Readiness Questionnaire (PAR-Q); and the MDS-UPDRS parts 1 and 2 (Goetz et al., 2008). We identified specific participant responses that would trigger follow-up examinations spanning both motor and non-motor symptoms of PD (i.e., the Freezing of Gait Questionnaire (FOG-Q) (Giladi et al., 2009), Parkinson's Disease Fatigue Scale (Grace et al., 2007), Activity Specific Balance Scale (ABC) (Dal Bello-Haas et al., 2011), and/or the Brief Pain Inventory (BPI)). For example, if a participant answered “mild”, “moderate” or “severe” to the MDS-UPDRS question 2.12 “Over the past week, have you usually had problems with balance and walking?” then they were automatically sent the ABC scale for further examination of balance confidence.

#### Considerations for Telehealth

Prior to administration of the examination battery, participants received an email providing information on session preparation (see Appendix). Instructions included technology specifications for telehealth sessions, access to questionnaires, overview of environmental setup, equipment needed for the session, care-partner involvement, and emergency action plans.

Prior to the telehealth session, assessors reached out to each participant to evaluate barriers and facilitators to safely conduct the examination. For example, we evaluated for the presence of a care-partner, fall or tripping hazards (i.e., rugs, wires, pets), access to a clear corner or stable surface for static balance assessments, and access to a clear narrow hallway for dynamic balance assessments (Table 1) (see Appendix for resources, considerations, and environmental checklists).

**Table 1.** Adaptation of Examination Battery for Telehealth

Examination Measures in Order of Administration	Type of Measure	Construct	Clinical Administration of Measure	Telehealth Adaptation of Measure
Physical Activity Readiness Questionnaire (PAR-Q) (Thomas etal., 1992)	Patient Reported Outcomes	Body Structure & Function, Activities	Pencil & Paper/Electronic	Electronic
Movement Disorders Society United Parkinson's Disease Rating Scale (MDS-UPDRS) Parts I & II (Goetz et al., 2008)	Patient Reported Outcomes	Body Structure & Function, Activities, Participation	Pencil & Paper/Electronic	Electronic
Parkinson's Fatigue Scale (Grace et al., 2007)	Patient Reported Outcomes	Body Structure & Function, Activities	Pencil & Paper/Electronic	Electronic
Freezing of Gait Questionnaire (FOG-Q) (Giladi et al., 2000)	Patient Reported Outcomes	Body Structure & Function, Activities, Participation	Pencil & Paper/Electronic	Electronic
Activities Specific Balance Confidence Scale (ABC) (Dal Bello-Haas et al., 2011)	Patient Reported Outcomes	Activities	Pencil & Paper/Electronic	Electronic
Brief Pain Inventory (BPI) - Short Form (Taghizadeh et al., 2022)	Patient Reported Outcomes	Body Structure & Function, Activities	Pencil & Paper/Electronic	Electronic
Movement Analysis of Tasks (Quinn et al., 2021)	Performance Based	Activities	In-person performance of 6 functional tasks (sitting, sit to stand, standing, walking, step up & down, reach/grasp/manipulate). These activities can be performed in an open clinic environment. The physical therapist is instructing, observing, guarding, and documenting performance.	Performance of 4–5 functional tasks (sitting, sit to stand, standing, walking, step up & down-if a step was available). Sitting and sit-to-stand were performed with a chair placed against a wall. Standing was performed with participant positioned in a corner with a solid support surface or care-partner in front of the participant for safety. Walking was performed in a narrow hallway where 2 walls could be used for support as needed. The physical therapist instructed the care-partner in safeguarding practices for these activities. The physical therapist was instructing, observing, and documenting performance throughout test administration.
5 x Sit to Stand (Duncan et al., 2011)	Performance Based	Body Structure & Function, Activities	A chair is placed up against a wall. The participant is seated in the chair. The PT has their hand on the chair and their foot against the leg of the chair so that it does not move. As the participant changes position, the therapist is guarding for safety and is observing/timing the performance.	A chair was placed against a wall. The participant began the test seated in the chair. The physical therapist instructed the care-partner to place their hand on the back of the chair and their foot against the leg of the chair so that it would not move during the test. As the participant changes position, the care-partner guarded the participant for safety and observed for any signs of difficulty or distress. The physical therapist observed and timed the participant's performance.
Timed Up & Go (TUG) Test (Brusse et al., 2005)	Performance Based	Activities	The physical therapist measures a 3-meter distance. A chair is placed at one end of the 3-meter walkway and a cone at the other end. The physical therapist instructs, guards throughout the duration of the test and times the performance.	Prior to the virtual session, the participant was instructed to place a chair against a wall, then measure a 3-meter distance from the base of the chair, marking the other end of the walkway with a piece of tape. The physical therapist instructed the care-partner in safeguarding practices for this activity. The physical therapist observed and timed the participant's performance on this task.
Timed Up & Go (TUG) Test – Cognitive (Maranhao-Filho et al., 2011)	Performance Based	Activities	See above description (TUG test). TUG was performed while participant was asked to count backwards from a random number (in the 90s) by 3. The number was provided to the participant after the word “go”.	See above description (TUG test). TUG was performed while participant was asked to count backwards from a random number (in the 90s) by 3. The number was provided to the participant after the word “go”.
Mini-BESTest (select components) (Leddy et al., 2011)	Performance Based	Body Structure & Function, Activities	All sub-categories (anticipatory, reactive postural control, sensory orientation, and dynamic gait) and test items of the miniBESTest are performed in-person, with a PT instructing, observing, guarding, and timing all activities. This is typically performed in an open space where participants have the ability for trial & error. If balance is questionable, additional safeguards such as testing within parallel bars of including additional guarding personnel may be used. For item #8 (feet together, eyes closed on foam), a dense foam pad is used in clinic.	Sub-categories were selected for telehealth examination and included: all anticipatory postural control items, select sensory orientation and dynamic gait items (see Figure 1.) Anticipatory balance activities items were performed with participant positioned in a corner with a solid support surface or care-partner who stood in front of the participant for safety. Dynamic gait activities were performed in a narrow hallway where 2 walls could be used for support as needed. The physical therapist instructed the care-partner in positioning of the recording devices and guarding for patient safety. The physical therapist also delivered instructions, observed participant performance and timed/scored test items. For item #8 (feet together, eyes closed on foam), participants were instructed to stand on a couch cushion.

#### Telehealth Assessment

We conducted a movement analysis of tasks including sitting, standing, sit to stand, walking and step up/down if appropriate. Participants were asked to sit and stand for 30 seconds each, and to perform one repetition of sit to stand, walking in a clear pathway (distance varied based on space availability) and step-up step-down using a stair with a railing. Task observations included speed, amplitude, symmetry, coordination, alignment, and postural control. Gait speed from a standing start was calculated for the walking task.

Results from the outcome measures and observations from the movement analysis of tasks prompted possible use of additional outcome measures including: 5 Times Sit to Stand (Duncan et al., 2011), Timed Up & Go (TUG) (Brusse et al., 2005), and TUG Cognitive (Maranhão-Filho et al., 2011), and several items from the mini-BEST including rise to toes, single leg stance, and stance (feet together) eyes open firm surface and eyes closed compliant (e.g. couch cushion) surface.^1^ Timed functional tests were timed by the physical therapist, unless there were internet connectivity issues, in which case the care-partner would time the participant. Virtual sessions were recorded, which allowed the evaluator to return to the evaluation and review components of testing as needed.

#### Trial Procedures in Healthy Adults

We first tested these examination procedures in a convenience sample of five healthy adults. The therapists evaluated metrics of accessibility to survey, accuracy of branching logic, and total time taken and feedback was provided by the participants. The examination procedures were then modified and finalized for implementation with PwP.

**Table 2.** Participant Demographics

	Case #1	Case #2	Case #3
Gender (M/F)	M	F	M
Education (yrs)	16	14	18
Race	White	White	White
Ethnicity	Non-Hispanic	Non-Hispanic	Non-Hispanic
Years since PD Dx (yrs)	2	25	20
H&Y Scale	III	III	III
Hx of recent falls	No	Yes	Yes
5 x STS (sec)	14.0	16.8	40.3
TUG (sec)	8.2	14.7	14.2
TUGcog (sec)	7.7	15.6	18.9
Gait Speed (m/sec)	1.1	0.4	0.5

*Note*. *Abbreviations*: M, Male; F, Female; yrs, years; PD, Parkinson Disease; Dx, Diagnosis; H&Y, Hoehn & Yahr; Hx, History; 5xSTS, 5 Times Sit to Stand; TUG, Timed Up and Go Test; TUGcog, Timed Up and Go Test Cognitive Version.

Participants completed demographic information and were screened for medical clearance to exercise using the PAR-Q. All participants passed the screening. Responses to questionnaires were reviewed by licensed physical therapists (CM, JF).

### Case 1 (C1)

#### Session Preparation

C1 had previously undergone telehealth examinations by medical professionals, including but not limited to physicians, speech therapy, and physical therapy. C1 connected to a telehealth session on a desktop computer with an accessory webcam device. An alternate device (iPhone 10, Apple, Cupertino, CA, USA) was also used for this assessment. A care-partner was present 100% of the time to provide assistance with technological and environmental set up, along with guarding for the examination. Assessment was performed in a large office (3.1 x 6.1 m), with hardwood floors, and a clear corner space available for static balance assessment, and in a long open hallway (5.8 m) with access to a staircase (ascending rail on right). All equipment was gathered, and the environment was set up for testing prior to assessment. Rugs were removed, window dressings were closed, and indoor lighting was used throughout testing for optimal visibility.

#### History and Systems Review

C1 was an 83-year-old cisgender male who racially identified as white, with a past medical history (PMHx) of high cholesterol controlled with atorvastatin, gastroesophageal reflux disease (GERD) controlled with Famotidine, autoimmune inner ear disease, abnormal thyroid hormone levels controlled with Levothyroxine, and arthritis controlled with Methotrexate. He was diagnosed with PD in 2020 based on neurologic examination. He had no family history of PD. C1 initially was diagnosed with essential tremor five years prior. However, symptoms of impaired balance and gait began in 2019 at which point the diagnosis of PD was considered. C1 reported that he was retired and lived in a two-story home in the suburbs with his wife (care-partner). Prior to diagnosis, he would exercise two to three times per week on a home elliptical. Despite attempts for continued home exercise, he experienced limited activity tolerance due to blood pressure irregularities from autonomic dysfunction.

#### Clinical Impression and Examination

Hoehn & Yahr score for C1 was stage III indicating moderate disease. Progressive symptoms of autonomic dysfunction combined with impaired balance and gait have led to decreased functional mobility and exercise tolerance. On review of questionnaire responses, C1 reported “slight” for items pertaining to pain, branching to receipt of the Brief Pain Inventory Short Form (Score = 5/10, moderate pain for lower back and left hip). On the MDS UPDRS Part II, C1 reported “slight” for items pertaining to impaired balance and walking branching to the ABC Scale (Score = 84.35%, balance confidence). Additionally, C1 reported “slight” for items pertaining to dizziness, fogginess, and feelings of faint, which clinically we wanted to evaluate further to determine if there were underlying vestibular pathologies, autonomic deficits, or other contributors to these sensations. Both low back pain and vestibular pathologies were investigated first by line of questioning, and second by formal evaluations (e.g. postural and biomechanical analysis, range of motion, and vestibular impairment-based testing). Movement analysis of tasks showed that C1 demonstrated impairments in motor control, balance, and efficiency of movement on tasks of sit to stand, stance, and walking. With the exception of prolonged stance, for which C1 reported mild dizziness/lightheadedness, none of the remaining activities provoked symptoms. Following the decision tree, the movement analysis of tasks supported continuation with all of the advanced motor testing with the exception of standing in tandem with eyes closed. C1 was successful at all other assessments.

#### Assessment

Testing revealed impairments in steady state and proactive balance with difficulty integrating sensory organization (proprioceptive and vestibular cues) along with symptoms of orthostasis. Combined, these findings contributed to decreased activity tolerance, instability in gait and decreased functional independence particularly for negotiation of community settings.

### Case 2 (C2)

#### Session Preparation

This was the first telehealth physical therapy encounter for participant C2. C2 connected using an iPad (Apple, Cupertino, CA, USA). The care partner was present 100% of the time to provide assistance with technological and environmental set up, along with guard assistance for assessments as needed. Assessment was performed on the ground floor of their 2-story private home. Most activities were completed in the foyer area (approximately 3m x 2.5m), which was connected to a long narrow hallway (11m), where the participant could reach both walls for stability if needed. A clear corner was available for static balance, as was a staircase with a right ascending railing for step negotiation. Hardwood floors were present throughout the assessment area. All requested equipment was collected prior to the start of the virtual session. Indoor lighting was used throughout and provided optimal visibility. The hallway lighting was limited, which made visibility challenging at times for the assessor.

#### History and Systems Review

C2 was a 73-year-old cisgender female, who racially identified as white, with a PMHx of Type II diabetes controlled with Januvia and Invokamet and hypertension controlled with Lisinopril. She was diagnosed with PD in 1997 based on neurologic examination. She reported no family history of PD. An initial symptom of upper extremity tremor was noticed by others. Current motor symptoms included tremor, dyskinesia, and balance difficulty, reporting two falls in the last year. C2 lived with her husband (care-partner) in a 2-story private home in the suburbs. She worked full-time as a teaching assistant until retiring in 2007. Prior to diagnosis, she was always busy with work, gardening, and other home activities. Her current activity includes walks with her husband and riding a 3-wheeled tricycle outdoors during the nice weather months. As her disease has progressed, C2 reported her balance becoming more problematic, requiring the use of a rollator walker for outdoor mobility tasks.

#### Clinical Impression and Examination

C2 presented at Hoehn & Yahr stage III indicating moderate disease severity, and progressive symptoms of imbalance have led to decreased physical activity. On the MDS-UPDRS I, C2 indicated “severe” nighttime sleep issues and “mild” daytime sleepiness branching to her receipt of the PD Fatigue Scale (score = 65/80, indicating fatigue severely impacts her daily activities). On the MDS-UPDRS II, C2 indicated “severe” problems with balance and walking, which branched to the ABC Scale (score = 40% balance confidence). She also reported “moderate” freezing of gait (FOG) when walking resulting in branching to the FOG-Q (score = 20/24, indicating FOG impacts daily activities). Movement analysis of tasks showed that C2 maintains static sitting without difficulty. Sit to stand was accomplished independently with increased weight bearing on her right lower extremity. She was able to maintain static standing for 30 sec without support, noting increased mediolateral sway. C2 walked 10m down a narrow hallway; she managed the distance without upper extremity support and without an assistive device. She was able to ascend and descend one step using a unilateral railing, using a step- to pattern and demonstrating poor motor control with ascent and descent. None of these activities provoked symptoms. Following the decision tree, movement analysis supported continuation with all of the advanced motor testing with the exception of 1-legged stance and standing on foam with feet together and eyes closed. She was successful at all other assessments.

#### Assessment

Testing revealed impairments in proactive balance and inability to maintain a 1-legged stance. Combined, these findings contributed to instability in gait and decreased functional independence particularly for negotiation of uneven surfaces and in community settings.

### Case 3 (C3)

#### Session Preparation

This was the first telehealth physical therapy encounter for participant C3. C3 connected using an iPad (Apple, Cupertino, CA, USA) and at times an iPhone (Apple iPhone, Cupertino, CA, USA). C3 used two devices for optimal viewing. Assessment was performed in the participant's home office (6m x 3.5m). The floor consisted of wall to wall low-pile carpeting. The room had furniture surrounding the outer edges, making the assessment space approximately 2.5 x 2.5m. A clear corner was not available for static balance assessment; thus, balance activities were performed using a chair back or desk-top for additional support if needed. Although stairs leading to a lower level were present, this participant's environment did not allow for camera viewing of this area, thus the step up and down component of movement analysis of tasks was not evaluated. All requested equipment was collected prior to the start of the virtual session. Indoor lighting was appropriate for assessor visibility.

#### History and Systems Review

C3 is a 53-year-old cisgender male, who racially identifies as white. He was diagnosed with PD in 2002 based on neurologic examination. C3 has no other significant PMHx and no family history of PD. C3 reports an initial symptom of tremor in both hands. Current motor symptoms include tremor, stiffness, dystonia, and balance difficulty, reporting two falls in the last year. C3 lives alone in a multi-level townhouse in the suburbs with his therapy dog. He has additional support from his girlfriend as needed. He works as a high school history teacher and a screenwriter. Prior to diagnosis, he was always active and enjoyed hiking and bicycling. He reports that his symptoms became progressively worse post diagnosis until he started an aggressive exercise program. His current activities include hiking, aquatherapy, yoga, and boxing. As his disease has progressed, he was impacted by bradykinesia, FOG, and dystonia, causing his ankle to turn in and has contributed to two or more falls in the past year.

#### Clinical Impression and Examination

C3 presents at Hoehn & Yahr stage III indicating moderate disease, however, he maintains a rigorous exercise routine. On review of questionnaire responses, C3 reported “moderate” for items pertaining to pain, branching to his receipt of the BPI Short Form (Score = 8/10, moderate pain for lower back, legs, feet, and head). On the MDS-UPDRS I, C3 indicated “moderate” nighttime sleep issues and “mild” daytime sleepiness branching to his receipt of the PD Fatigue Scale (score = 51/80, indicating fatigue moderately impacts his daily activities). On the MDS-UPDRS II, C3 indicated “severe” problems with balance and walking, branching to the ABC Scale (score = 39.3% balance confidence). Movement Analysis of Tasks showed that C3 maintains static sitting without difficulty. He was able to transition from sit to stand without use of upper extremity support but had difficulty on subsequent repetitions as his typical method is to use both arms and push off his thighs when rising to stand. His movement was segmental and not fluid. He used momentum and excessive trunk flexion to achieve adequate forward posture to achieve standing. He was able to maintain static standing for 30 sec without support (eyes open and eyes closed), with increased overall sway with eyes closed. C3 walked 3.93 meters on plush carpet, with a slightly increased base of support. Gait speed was very slow at 0.47m/sec. He was unable to complete the assessment of stairs as C3's home did not have stairs. Following the decision tree, the movement analysis of tasks supported continuation with all of the advanced motor testing with the exception of all standing activities on uneven surfaces. He was successful at all other assessments.

#### Assessment

Testing revealed difficulty with sit to stand due to bilateral lower extremity weakness and impairments in motor control. Combined, these findings contributed to challenges with transfers from soft and low surfaces. Difficulty with transfers and slow gait speed put him at increased risk of falls and create challenges with negotiation of some community settings.

**Figure 2.**
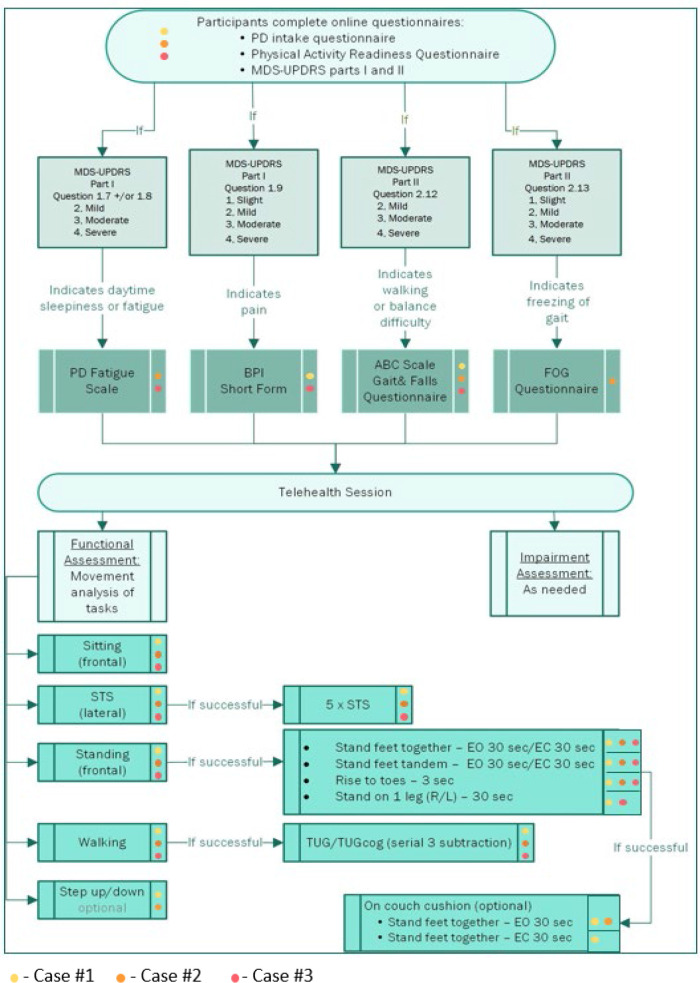
Clinical Decision Tree Including Colored Dots Representing the Individual Path of Each Participant

### Metrics of Feasibility

We evaluated feasibility of the telehealth physical therapy battery (Table 3) including both participant and clinician metrics (i.e., accuracy of survey branching logic, time taken to complete surveys vs examination, efficiency of examination, etc.). The length of time taken to complete the surveys and examination was reasonable across the medical complexities of the three participants. Survey time ranged between 10–20 minutes, while the examination ranged between 45–90 minutes. Environmental limitations (i.e., configuring the environment safely for planned testing, or additional testing), or technological limitations (i.e., connectivity, device set-up) delayed the physical therapy examination. The survey battery appeared to enhance planning for the physical therapy evaluation, leading to efficiency with data collection.

**Table 3.** Metrics of Feasibility

***Patient Facing Metrics***	
**Accuracy of branching logic**	100% accurate.
**Delivery of survey**	All 3 participants were able to access the survey without difficulty.
**Length of time to respond to the survey request**	If requested by the therapist directly - 1 day response time. If requested by admin/study team unknown to patient - up to 1 week response time.
**Length of time for survey completion**	Minimum 10 min. - maximum 20 min. (needed if follow-up surveys were sent).
**Presence of care partner**	C1 - 100% of time had care partner. C2 - 100% of time had care partner. C3 - 0% of time had care partner.
**Environmental limitations**	C1 - none C2 - Chair height not suitable, participant's feet did not touch the floor when seated due to short stature. Participants received the pre-assessment set up instructions, however, the environment was not set up for assessment at time of evaluation (Delay 5–10 minutes). C3 - Could not access stairs for Movement Analysis of Tasks. Participants received the pre-assessment set up instructions, however, the environment was not set up for assessment at time of evaluation (Delay 5 minutes).
**Technological difficulties**	C1 - Connecting to Zoom on alternate devices, negotiating webcam to provide accurate view of participant for evaluation (Delay 15 minutes). No call interruptions or sound/video quality issues throughout assessment. C2 - No problems on telehealth with care-partner present. No call interruptions throughout assessment, however due to the location of the participant in the room during assessment, care-partner needed to relay test instructions and reiterate participant's responses due to participants heanng limitation, and hypophonia. C3 - Connecting to an alternate device for movement analysis (Delay 5 minutes). No call interruptions, or sound/video quality issues throughout assessment.
**Clinician Facing Metrics:**	
**Session time saved with advanced survey administration**	15–30 minutes for completion of survey by participant, and review of survey responses by clinician.
**Length of time for completion of telehealth visit**	C1 - 90 minutes (medically complex, technological difficulties). C2 - 60 minutes (10 minutes for environmental set-up). C3 - 45 minutes (10 minutes for environmental set up).

## Discussion

In this three-case exemplar, we demonstrated the feasibility of conducting a comprehensive physical therapy examination via telehealth by following a decision tree to facilitate clinical decision making. The three participants demonstrated the heterogeneity of PwP, as all were mid stage (Hoehn & Yahr stage III) but had varying degrees of medical complexity and different clinical presentations. The decision tree was adaptable to each case presentation, creating individual pathways for examination based on their presenting signs and symptoms. In particular, the questionnaires on the decision tree offered clinicians opportunities to explore additional lines of questioning during history and systems review, whereas the movement analyses offered a chance to explore baseline movement capabilities and branch out to assess more challenging tasks and eliminate tasks that seemed unattainable.

The number of people diagnosed with PD is on the rise. Annually, close to 90,000 people in North America are diagnosed with PD, and there is a projected prevalence of 1,238,000 by 2030 (Dorsey et al., 2018). This projected growth requires a healthcare system that keeps pace with it, including increasing the number of physical therapists who are knowledgeable and confident in the examination and treatment of PwP. Recent publications have provided recommendations for examination (Kegelmeyer et al., n.d.) and intervention (Osborne et al., 2022) for PwP, however there is little focus on modifications required to conduct examinations via telehealth. Our proposed examination battery combines the PD-EDGE with additional outcome measures and examination items that are feasible to be conducted via telehealth.

An important aspect of the physical therapy examination is the interview, where therapists understand a patient's current therapy concerns and functional goals. This personal understanding facilitates communication between patient and therapist, builds rapport and trust, and it has the potential to increase patient compliance as well as greater outcome success (Diener et al., 2016). Obtaining survey data prior to the virtual visit allows therapists to be more efficient with interview time, by developing focused, personalized discussion points.

Although many physical therapy interventions for PwP are amenable to telehealth, development of an individualized multi-modal intervention plan requires a comprehensive examination battery to facilitate clinical decision making, design the plan of care, and evaluate patient progress (Flynn et al., 2023; Osborne et al., 2022; Quinn et al., 2020; Shih et al., 2022; van der Kolk et al., 2019). One of the primary concerns in telehealth is safety of administering tests and measures when a therapist is not in person to provide guarding and environmental cueing (Bennell et al., 2021; Bianchini et al., 2022; Colón-Semenza et al., 2023; Cubo et al., 2021; Thwaites et al., 2023). While there are a few outcomes that do not lend themselves well to telehealth administration (i.e., compensatory stepping, 6MWT), many movement-based assessments can readily be adapted to telehealth administration. Discussion regarding the preparation of the environment and technology ahead of time, along with care-partner participation, enables the physical therapist to implement safe and efficient procedures for telehealth examinations.

The telehealth examination offers the opportunity for a clinician to evaluate not only the participant and their needs, but inter- and intra-personal, social, and environmental barriers and facilitators for potential intervention efficacy (Kava et al., 2020). A key aspect of skilled physical therapy is a therapist's ability to implement functional activities that are challenging to the patient, yet equally attainable (Schenkman et al., 2018; Sparrow et al., 2016). A physical therapist's clinical decision making and judgment impact selection of activities. For a telehealth physical therapy program to be successful, physical therapists should be encouraged to provide appropriate challenges in a safe, structured manner.

## Study Limitations

This case series included a sample of convenience of participants who were known to the examiners. Although this is a small case exemplar, we recognize that our sample was not racially or ethnically diverse, despite medical diversities. Our study aimed to produce a comprehensive physical therapy examination battery for PwP. However, at this time many tests and measures recommended for use in PwP have yet to be validated for administration on telehealth, and further, there are known environmental constraints that make some tests and measures inappropriate for use within a telehealth setting (i.e., space limitations for the 6mWT, or 10mWT; space, materials or trained personnel for administration of the mini-BESTest). Ultimately these factors limit our ability to provide formal recommendations to therapists moving forward.

## Conclusion

A comprehensive examination battery lays the foundation to facilitate clinical decision making, design the plan of care, and evaluate patient progress. This physical therapy examination battery with embedded decision tree and branching logic provides physical therapists with a method to design and conduct safe, appropriately challenging, and efficient telehealth examinations in PwP. Future directions include determining the reliability and feasibility of this examination battery across a larger sample of PwP with different stages of Hoehn & Yahr, and inclusion in an intervention trial to determine impact on intervention planning.

## Footnotes

1 The email instructions to participants included determining a location for static balance testing (i.e., corner space, with a stable object or surface to regain balance if lost), dynamic balance testing (i.e., chair against a wall to avoid retropulsion for 5 Times Sit to Stand), and how to measure distances for completion of the TUG Test.

## Author Notes

**Disclosure of any conflicts of interest:** The authors of this report have no conflicts of interest to disclose.

**Sources of Funding:** None

**Previous presentations:** Part of this work was presented at the American Physical Therapy Association's Combined Sections Meeting in 2022.

## Appendix

Safety Considerations for In-Home Evaluations:

**Table:**

Environmental Requirements	Free standing corner (could be in a room, or corner of kitchen counters) Hallway or kitchen with countertop on either side Standard height chair (not on wheels) positioned against a wall for stability Access to a step or stairs if applicable/appropriate Additional chairs positioned throughout testing area in the event of fatigue, or low blood pressure Clean dry floors, free from loose cords, wires, rugs Good lighting Appropriate background (i.e., your patient is not positioned in front of a window)
Technology Requirements & Accessories	2 devices with live video capture quality (i.e., desktop with webcam, laptop with built in webcam, tablet, smart phone) Accessory battery or charging station Tripod or device stand Adjustable camera angles
Support Network (Care-partner, friend or family member)	Do the care-partner/patient understand how to use the technology you will be working with? If not, you may need to send information ahead of time, or perform a tutorial. Supervision or Guarding Environment or Task Set Up Technical troubleshooting Medical Safety Device setup, manage camera, allowing observation during examination
Additional Materials	Paper/Pen Towels/Blankets Pillows Assistive Devices (i.e., cane, walker, rollator, etc.) Blood pressure cuff
Emergency Plan (EAP)	Prior to examination, obtain: Primary Care Physician's name and contact information Neurologist's name and contact information The closest Emergency Services Department or hospital of choice to the participant Emergency Contact list Explain EAP to the participant, their care-partners, and emergency contacts (if different). Have a cellphone or landline nearby the participant and/or examination area Care-partner training hand-out prior to interaction with post-test to ensure completion Therapist will notify all parties that the examination is taking place on X date, at X time. In the event of a medical emergency: If a call is received from the treating therapist during the designated examination period, there is an emergency. The therapist will explain what has occurred, the emergency contact will then call 911 with this information to get the participant the fastest service. The therapist will remain on the telehealth call with the participant until EMS arrives. The therapist will explain events and will provide a record of the event to the participant's PCP, and Neurologist.

### Links to Additional Telehealth Resources:

Environmental Set-Up: Sample Introductory Email to Participants

APTA's Position on Telehealth: APTA Position Paper: Expanded Telehealth Access Act of 2023

APTA Telehealth Certificate Series: https://aptatherapists.elevate.gocadmium.com/p/TH-CERT

Telehealth Readiness Questionnaire: http://www.ncfh.org/uploads/3/8/6/8/38685499/final_patient_telehealth_readiness_tool_.pdf#:~:text=NCFH%E2%80%99s%20Patient%20Telehealth%20Readiness%20assessment%20tool%20is%20intended,their%20patient%20population%20by%20providing%20the%20necessary%20support

Preparing for a telehealth visit (clinician and patient): https://telehealth.hhs.gov/providers/preparing-patients-for-telehealth

Tips on performing a safe and efficient telehealth evaluation: https://thenonclinicalpt.com/telehealth-physical-therapy/#the-evaluation

Planning to maximize efficiency within telehealth sessions: https://telehealth.hhs.gov/providers/planning-your-telehealth-workflow

Risk management considerations in Telehealth and Telemedicine - HPSO: https://www.hpso.com/Resources/Telehealth-Telemedicine/Risk-Management-Considerations-in-Telehealth-and-T

VA resources including modifying the environment and various intervention types: http://www.rstce.pitt.edu/varha/

Updated new on telehealth policies and resources for regions of the US: https://telehealthresourcecenter.org/

Publication Reviewing Tips for a Safe and Successful Telehealth session: Havran MA, Bidelspach DE. (2021). Virtual physical therapy and telerehabilitation. Physical Medicine and Rehabilitation Clinics of North America. 32(2):419–428. https://doi.org/10.1016/j.pmr.2020.12.005

CDC STEADI - Older Adult Fall Prevention Materials for Patients and Care-Partners: https://www.cdc.gov/steadi/patient.html
